# New Surgical Criteria for Intraductal Papillary Mucinous Neoplasm Based on the Age-Adjusted Charlson Comorbidity Index Values and Presence of Solid Component

**DOI:** 10.3390/diagnostics14222582

**Published:** 2024-11-17

**Authors:** Hiroyuki Hasegawa, Mitsuharu Fukasawa, Shinichi Takano, Satoshi Kawakami, Natsuhiko Kuratomi, Shota Harai, Dai Yoshimura, Naoto Imagawa, Tetsuya Okuwaki, Toru Kuno, Yuichiro Suzuki, Takashi Yoshida, Shoji Kobayashi, Mitsuaki Sato, Shinya Maekawa, Naohiro Hosomura, Hiromichi Kawaida, Daisuke Ichikawa, Nobuyuki Enomoto

**Affiliations:** 1 Department of Gastroenterology, Faculty of Medicine, University of Yamanashi, 1110, Shimokato, Chuo 409-3898, Yamanashi, Japan; hirohasegawa@yamanashi.ac.jp (H.H.); stakano@yamanashi.ac.jp (S.T.); skawakami@yamanashi.ac.jp (S.K.); nkuratomi@yamanashi.ac.jp (N.K.); sharai@yamanashi.ac.jp (S.H.); dyoshimura@yamanashi.ac.jp (D.Y.); nimagawa@yamanashi.ac.jp (N.I.); tokuwaki@yamanashi.ac.jp (T.O.); tkuno@yamanashi.ac.jp (T.K.); yuichirohs@yamanashi.ac.jp (Y.S.); tyoshida@yamanashi.ac.jp (T.Y.); shoji@yamanashi.ac.jp (S.K.); satom@yamanashi.ac.jp (M.S.); maekawa@yamanashi.ac.jp (S.M.); enomoto@yamanashi.ac.jp (N.E.); 2 Department of Gastroenterology, Japan Community Health Care Organization, Yamanashi Hospital, 3-11-16, Asahi, Kofu 400-0025, Yamanashi, Japan; 3 Department of Surgery, Japan Community Health Care Organization, Yamanashi Hospital, 3-11-16, Asahi, Kofu 400-0025, Yamanashi, Japan; naohiro.955@gmail.com; 4 First Department of Surgery, Faculty of Medicine, University of Yamanashi, 1110, Shimokato, Chuo 409-3898, Yamanashi, Japan; kawaidah@yamanashi.ac.jp (H.K.); dichikawa@yamanashi.ac.jp (D.I.)

**Keywords:** intraductal papillary mucinous neoplasm, high-risk stigmata, solid component, age-adjusted Charlson comorbidity index

## Abstract

**Objectives**: The present study aimed to validate the new international guidelines for IPMN and determine the surgical criteria for patients with IPMN exhibiting high-risk stigmata (HRS). **Methods**: We enrolled 115 IPMN patients exhibiting HRS who were diagnosed between 2004 and 2021. Of the 115 patients, 79 underwent surgery (surgical group) and 36 did not undergo surgery (non-surgical group). The overall survival (OS) of each group was compared, and multivariate analysis was performed to identify factors associated with OS. **Results**: There was no significant difference in the estimated 5-year OS in the surgical and non-surgical groups (67% vs. 74%; *p* = 0.75). The presence of a solid component (SC) (hazard ratio [HR], 6.92; 95% confidence interval [CI], 3.30–14.5) and a high score of age-adjusted Charlson comorbidity index (ACCI) (≥5) (HR, 2.27; 95% CI, 1.11–4.64) were independent predictors of poor OS. In the presence of an SC, the surgical group had a significantly better OS than the non-surgical group (estimated 5-year OS, 38% vs. 18%; *p* = 0.031). In the absence of an SC, the prognosis of patients with a high ACCI was significantly poorer than those with a low ACCI in the surgical group (estimated 5-year OS, 59% vs. 93%; *p* = 0.005). **Conclusions**: An SC and a high ACCI are important prognostic factors in IPMN patients exhibiting HRS. Thus, patients with an SC should undergo surgical resection. However, conservative management may be the optimal treatment in patients without an SC and with a high ACCI.

## 1. Introduction

Intraductal papillary mucinous neoplasm (IPMN) of the pancreas is characterized microscopically as papillary growth of columnar neoplastic cells with mucin hypersecretion and morphologically as cystic dilation of the excretory pancreatic duct. IPMN is a precursor of pancreatic cancer [1,2,3]. IPMN can be graded pathologically as low-grade dysplasia (LGD), high-grade dysplasia (HGD), and IPMN-derived invasive carcinoma (IC), and progression of IPMN is recognized to follow from LGD to HGD/IC (adenoma-carcinoma sequence). Surgical resection is recommended for malignant IPMN, such as IC and HGD. The revised international consensus guidelines for IPMN 2017 established high-risk stigmata (HRS) as predicting IC and HGD. HRS is defined as obstructive jaundice in a patient with cystic lesions of the pancreatic head, enhanced mural nodules ≥ 5 mm, or a main pancreatic duct (MPD) diameter of ≥10 mm [4]. The recently released international evidence-based Kyoto guidelines for IPMN (Kyoto guidelines 2024) added a solid component (SC), which represents IC, to the conventional HRS. According to the Kyoto guidelines 2024, patients with HRS are considered for surgical resection on the basis of their surgical tolerance [5]. The increased use of and recent advances in imaging modalities have led to the frequent identification of incidentally diagnosed IPMN, especially in the elderly [6]. Although pancreatic surgery is feasible for elderly patients [7], postoperative mortality and morbidity are relatively high [8,9]. Because elderly patients may have serious comorbidities, in the clinical setting, some patients with IPMN exhibiting HRS are observed for long periods without undergoing surgical resection [10]. A meta-analysis showed that IPMN-related mortality among patients with HRS who did not undergo surgery due to higher age or comorbidities was low, and the risk of death from other causes was much higher [11]. Thus, further clarification is required regarding which patients with IPMN exhibiting HRS should undergo surgical resection and which should be followed up due to advanced age and serious comorbidities.

The Kyoto guidelines 2024 state that the decision for surgical resection should be based on the degree of suspicion of IC/HGD and the patient’s general condition, comorbidity, and life expectancy [5]. However, the ability of the conventional HRS definition, as a surgical indication criterion, to predict IC/HGD is unsatisfactory [12,13,14,15], and the clinical impact of the new definition is yet to be evaluated. In addition, specific criteria with respect to the patient’s general condition, comorbidities, and life expectancy have not been defined [5]. The age-adjusted Charlson comorbidity index (ACCI) is often used to estimate the prognosis based on the patient’s comorbidities before treatment. ACCI is an established predictor of poor postoperative prognosis in various cancers, including pancreatic cancer [16,17,18,19]. However, the relationship between the ACCI and the prognosis of IPMN patients with HRS is not yet well understood.

In the present study, we aimed to clarify the appropriate surgical criteria by comparing the survival outcomes of surgical and non-surgical treatment for patients with IPMN exhibiting HRS and identifying the factors associated with survival.

## 2. Methods

### 2.1. Study Design and Patients

This single-center, retrospective, observational study included patients with IPMN exhibiting HRS who were diagnosed at Yamanashi University Hospital between January 2004 and June 2021. The patients had no distant metastasis and had complete data that was worthy of this research. The patients were also divided into surgical and non-surgical groups depending on whether they had undergone surgery or not. The exclusion criteria were as follows: pancreatic ductal adenocarcinoma concomitant with the IPMN and a follow-up of <6 months unless the patients died within that time period. This study was conducted in accordance with the principles of the Declaration of Helsinki, and it was approved by the Institutional Review Board for Clinical Research at Yamanashi University Hospital.

### 2.2. Classification of IPMN

The diagnosis and morphological classification of IPMN were made in accordance with the Kyoto guidelines 2024. Main duct IPMN (MD-IPMN) was defined as segmental or diffuse dilation of MPD of ≥5 mm without other causes of obstruction, and branch duct IPMN (BD-IPMN) as pancreatic cysts of ≥5 mm in diameter that communicate with the MPD. Mixed IPMN met the criteria for both MD-IPMN and BD-IPMN [5]. Diagnosis of IPMN was based on the findings of any one of these imaging modalities: computed tomography (CT), magnetic resonance imaging with magnetic resonance cholangiopancreatography, and endoscopic ultrasonography (EUS).

### 2.3. Age-Adjusted Charlson Comorbidity Index: ACCI

We used ACCI to quantify the baseline comorbidities. Patients with comorbidities were scored first using the Charlson comorbidity index (CCI). CCI is a weighted scoring system that incorporates 19 different medical categories, each of which is weighted based on its potential impact on mortality. The ACCI was calculated by weighting the individual CCI and adding 1 point per decade to ages > 40 years (points: 1, for 50–59 years; 2, for 60–69 years; and 3, for 70–79 years). The predicted 10-year survival rates according to each ACCI score in the testing population are as follows: 99%, score 0; 96%, 1; 90%, 2; 77%, 3; 53%, 4; and 21%, 5 [20]. The cut-off value for the ACCI was established by performing receiver operating characteristic (ROC) curve analysis. In the present study, an ACCI score of ≥ the cut-off value was defined as high ACCI, and was assessed as a prognostic factor for OS.

### 2.4. Solid Component: SC

**Figure 1:**
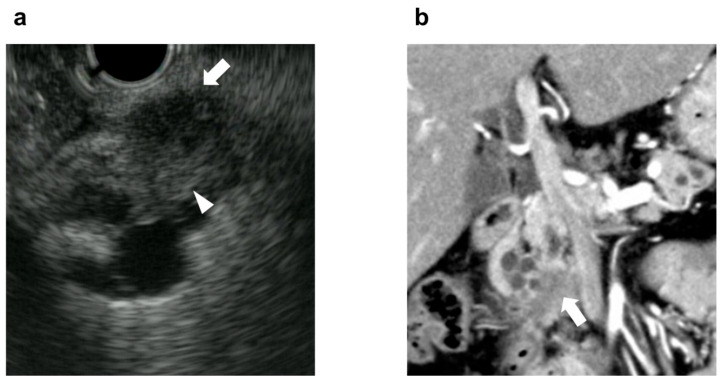
In this study, an irregular mass in the pancreatic parenchyma that was contiguous with a mural nodule was considered an SC. (**a**) EUS showing an SC (arrow) in the pancreatic parenchyma that is contiguous with the mural nodule (arrowhead). (**b**) CT showing an SC (arrow) infiltrating the pancreatic parenchyma from within the cyst. SC, solid component; EUS, endoscopic ultrasonography; CT, computed tomography.

The Kyoto guidelines 2024 add SC to the conventional HRS, and state that an SC is a mass in the pancreatic parenchyma and indicates the possible presence of IPMN with IC [5]. In this study, an irregular mass in the pancreatic parenchyma that was contiguous with a mural nodule on EUS or CT was considered an SC according to previous reports (Figure 1) [21,22,23,24,25,26,27].

### 2.5. Outcomes

The primary outcome of the present study was a comparison of patient survival between the surgical and non-surgical groups. We compared the overall survival (OS) and disease-specific survival (DSS) between the surgical and non-surgical groups. OS was defined as the period from pancreatectomy or HRS diagnosis to death due to any cause. DSS was defined as the period from pancreatectomy or HRS diagnosis to IPMN-related death. Secondary outcomes were factors associated with survival in patients with IPMN exhibiting HRS. To elucidate the predictors for OS in patients with IPMN exhibiting HRS, the clinical characteristics were compared in a univariate analysis. Subsequently, multivariate analysis was performed to identify the factors independently associated with OS using those variables found to be statistically significant on univariate analysis. The following clinical characteristics were assessed: age, sex, ACCI, IPMN type, MPD diameter, mural nodule height, presence of jaundice, presence of an SC, and pancreatectomy.

### 2.6. Statistical Analyses

The continuous variables were analyzed using the Mann–Whitney U test, and the categorical variables were analyzed using the chi-square for independence test or Fisher’s exact test. OS and DSS were measured using the Kaplan–Meier method, and the survival estimates were compared using the log-rank test. A Cox proportional hazards model was used to perform the univariate and multivariate analyses to determine the significant predictors of OS. A *p*-value of <0.05 was considered statistically significant.

All statistical analyses were performed with EZR (Version 1.52; Saitama Medical Center, Jichi Medical University, Saitama, Japan), which is a graphical user interface for R (The R Foundation for Statistical Computing, Vienna, Austria) [28]. More precisely, it is a modified version of R Commander (Version 2.7-0) designed to add the statistical functions frequently used in biostatistics.

## 3. Results

### 3.1. Characteristics of Included Studies

This study enrolled 120 patients with IPMN exhibiting HRS. A flow diagram of the study population is presented in Figure S1. We excluded one patient with concomitant PDAC and four patients with an observation period of <6 months. Finally, 115 patients were included in this study. Of the 115 IPMN patients, 79 underwent surgery (surgical group), and 36 did not undergo surgery (non-surgical group) due to advanced age or comorbidities.

The median follow-up period in all the patients was 1256 days (3 years and 5 months). The clinical characteristics of the surgical and non-surgical groups are included in Table 1. There were no significant differences in most of the clinical characteristics, including sex, IPMN type, MPD diameter, mural nodule height, presence of jaundice, presence of an SC, and the proportions of cause of death between the surgical and non-surgical groups. However, the median age of the non-surgical group was higher than that of the surgical group (81 years vs. 72 years, *p* < 0.001) and the median ACCI of the non-surgical group was higher than that of the surgical group (5 vs. 4, *p* = 0.001). The pathological diagnoses in the surgical group were as follows: LGD (*n* = 29, 37%), HGD (*n* = 21, 26%), and IC (*n* = 29, 37%). Of the 37 patients who died during the follow-up, 19 (16.5%) died due to IPMN, and 12 (10.5%) died from a cause unrelated to IPMN: three cases of other organ cancer, two of aspiration pneumonia, one of cardiovascular disease, one of cholangitis, one of alcoholic liver cirrhosis, one of liver abscess, one of uremia, one of duodenal hemorrhage, and one of lung hemorrhage (Table S1). The range of the ACCI in the entire cohort was 0–11 with a median of 4. The cut-off value for the ACCI was five based on the ROC curve (Figure S2). An ACCI score of ≥5 was defined as high ACCI, and this was assessed as an associated factor for OS.

In the surgical group, an SC was preoperatively diagnosed in 29 patients. The histopathological results of the 29 patients were as follows: LGD in one patient (3%), HGD in four patients (14%), and IC in 24 patients (83%). The histopathological results of the 50 patients without an SC were as follows: LGD in 28 patients (56%), HGD in 17 patients (34%), and IC in 5 patients (10%). The SC demonstrated 83% sensitivity, 90% specificity, 83% positive predictive value (PPV), and 90% negative predictive value (NPV) for diagnosing IC. The majority of cases that could not be diagnosed preoperatively were minimally invasive carcinomas, and the invasive areas could not be recognized as SCs. The representative EUS and pathological findings of IC are presented in Figure S3.

### 3.2. Comparison of Patient Survival Between the Surgical and Non-Surgical Groups

**Figure 2:**
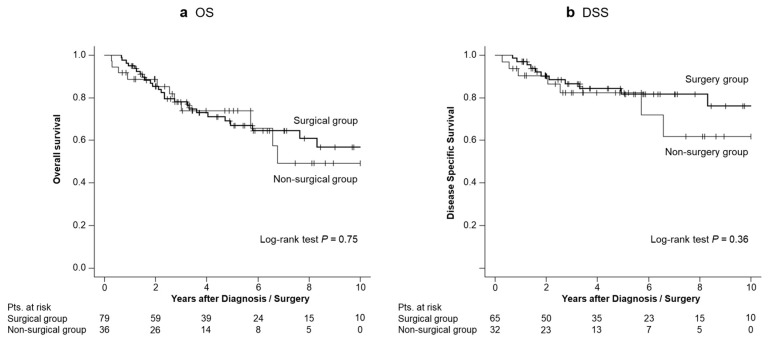
Overall survival (OS) and disease-specific survival (DSS) in the entire cohort. (**a**) The estimated 5-year OS in the surgical and non-surgical groups were 67% and 74%, respectively (*p* = 0.75). (**b**) The estimated 5-year DSS in the surgical and non-surgical groups were 82% and 82%, respectively (*p* = 0.36).

There was no statistically significant difference in the estimated 5-year OS between the surgical and non-surgical groups (67% vs. 74%, *p* = 0.75; Figure 2a). Furthermore, there was no statistically significant difference in the estimated 5-year DSS between the surgical and non-surgical groups (82% vs. 82%, *p* = 0.36; Figure 2b).

### 3.3. Factors Associated with Survival in Patients with IPMN Exhibiting HRS

Univariate analysis showed age (*p* = 0.018), a high ACCI (*p* < 0.001), presence of jaundice (*p* = 0.028), and presence of an SC (*p* < 0.001) were associated with a poor OS in patients with IPMN exhibiting HRS. In a multivariate analysis model including the above four variables, which were significant in univariate analysis, the presence of an SC (hazard ratio [HR], 6.92; 95% confidence interval [CI], 3.30–14.5) and a high ACCI (HR, 2.27; 95% CI, 1.11–4.64) were identified as independent prognostic factors. In contrast, pancreatectomy and the factors of the conventional HRS definition (MPD diameter, mural nodule height, and presence of jaundice) were not statistically associated with prognosis (Table 2).

### 3.4. Survival Analyses Stratified by the Presence or Absence of an SC

**Figure 3:**
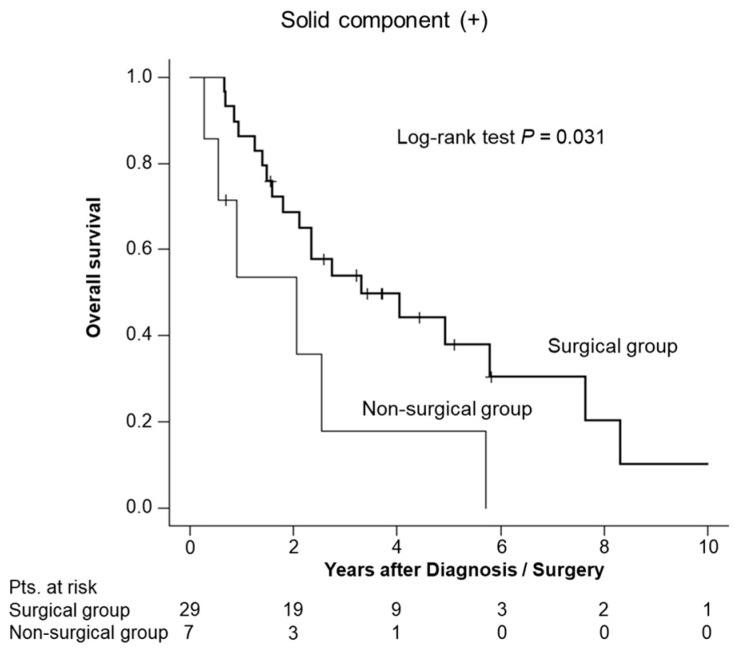
Overall survival (OS) in patients with a solid component. The estimated 5-year OS in the surgical and non-surgical groups were 38% and 18%, respectively (*p* = 0.031).

**Figure 4:**
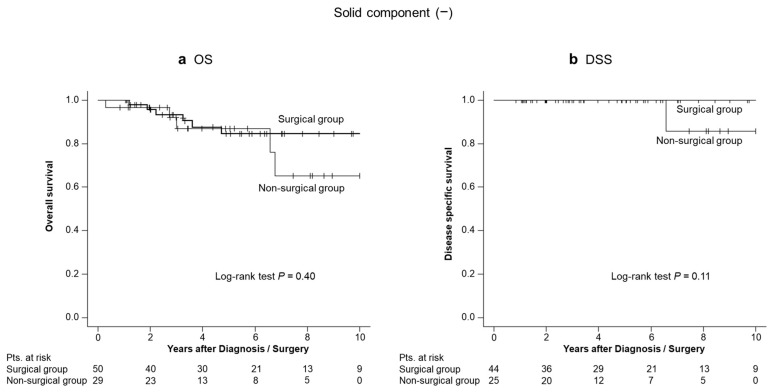
Overall survival (OS) and disease-specific survival (DSS) in patients without a solid component. (**a**) The estimated 5-year OS in the surgical and non-surgical groups were 85% and 87%, respectively (*p* = 0.40). (**b**) There was only one IPMN-related death, and the estimated 5-year DSS was 100% in both groups (*p* = 0.11).

To evaluate the impact of an SC on OS, we compared the OS between the surgical and non-surgical groups, stratified by the presence or absence of an SC. In the presence of an SC, the surgical group had a significantly better OS than the non-surgical group (estimated 5-year OS, 38% vs. 18%; *p* = 0.031; Figure 3). In the absence of an SC, there was no statistically significant difference between the surgical and non-surgical groups (estimated 5-year OS, 85% vs. 87%; *p* = 0.40 (Figure 4a); estimated 5-year DSS was 100% in both groups; *p* = 0.11 (Figure 4b)). Among the patients without an SC, only one patient died due to IPMN. In this patient, the SC did not present at the time of HRS diagnosis; it appeared 6 years and 1 month later. Five months after the SC detection, the patient died due to IPMN.

In the absence of an SC in the surgical group, patients with a high ACCI had a significantly poorer OS than those with a low ACCI (estimated 5-year OS, 59% vs. 93%; *p* = 0.005; Figure S4).

## 4. Discussion

In this study of IPMN patients with HRS, two important clinical findings were obtained. First, there was no significant difference in OS between the surgical and non-surgical groups. Second, the presence of an SC and a high ACCI were independent factors predicting a poor OS.

The Kyoto guidelines 2024 state that patients with HRS are considered for surgical resection. In addition to this statement, the current guidelines also recommend decisions regarding operative indication be made carefully, based not only on the degree of suspicion of HGD/IC but also on the patients’ general condition, comorbidity, and life expectancy [5]. Most patients diagnosed with IPMN are older adults and they often have some comorbidities. In clinical settings, although some patients with IPMN exhibiting HRS do not undergo surgical resection due to age or serious comorbidities, the patients who are observed for long periods among such patients are not very few. Crippa et al. reported a 5-year DSS rate of 60.2% among 50 IPMN patients with HRS who did not undergo surgical resection [29]. Sakai et al. analyzed a cohort of 101 IPMN patients exhibiting HRS who did not undergo surgical resection in a multicenter, retrospective study. They demonstrated that neither mural nodules ≥ 5 mm nor MPD ≥ 10 mm were related to a worse prognosis, with estimated 5-year OS and DSS rates of 74% and 91%, respectively [10]. In this study, the prognosis of patients with IPMN exhibiting HRS was as good as the previous reports, with estimated 5-year OS and DSS rates of 74% and 82%, respectively, in the non-surgical group. To the best of our knowledge, this study is the first to compare the prognosis of patients with IPMN exhibiting HRS between the surgical and non-surgical groups. There was no significant difference in OS between the surgical and non-surgical groups. This may be due to the fact that HRS, which was indicated for surgery in the Kyoto guidelines 2024, did not adequately predict malignant IPMN and the high number of IPMN-unrelated deaths. In this study, only 37% of the patients with IPMN who underwent surgery due to HRS showed IC, while the remaining 63% had non-invasive lesions. Several studies have reported that the conventional HRS definition can diagnose malignant IPMN with a 59–90% sensitivity and 67–79% specificity [12,13,14]. These results indicate that the decision regarding surgery should not only depend on HRS and that new surgical criteria are required.

The Kyoto guidelines 2024 define SC, a mass in the pancreatic parenchyma, as a new HRS in addition to the conventional HRS [5]. In the present study, we included the presence of an SC among the variables and performed univariate and multivariate analyses to identify the factors independently associated with OS. This study demonstrated that neither pancreatectomy nor the factors of the conventional HRS definition were related to poor prognosis and that the presence of an SC and a high ACCI were independent factors predicting a poor OS. In cases with SC, the surgical group had a better prognosis than the non-surgical group. An SC indicates the possible presence of an IC. The PPV and NPV of SC to IC were 83% and 90%, respectively, which were as superior as in previous reports (86–100% PPV, 79–94% NPV) [25,26]. These results suggest that IPMN patients with an SC are likely to have invasive cancer, and aggressive surgical resection is recommended to improve prognosis.

In patients without an SC, there were no IPMN-related deaths within 5 years of surgery or HRS diagnosis in either the surgical or non-surgical groups. Based on its ability to predict IC, IPMNs without an SC are unlikely to be IC. Thus, the short-term risk of IPMN-related mortality is very low, even without surgery. The ACCI, modified by adding age to the CCI, is a tool to assess a patient’s comorbidities and predict mortality risk. The patients with a high ACCI had a poorer prognosis than those with a low ACCI in the surgical group. A high ACCI is an established predictor of poor postoperative prognosis in various cancers, including pancreatic cancer [16,17,18,19], and surgical intervention should be carefully considered in these patients. Furthermore, some previous studies highlighted the need for taking the life expectancy according to ACCI into consideration for IPMN surveillance. Sahora et al. assessed 725 IPMN patients who underwent surgical resection or have been followed up, and found that the median survival time for all patients with ACCI ≥ 7 was 43 months compared with 180 months in patients with lower ACCI. They also revealed that the chance of IPMN-unrelated death within 3 years of diagnosis was 11-fold higher for patients with ACCI of ≥7 than for patients with lower scores [30]. Likewise, Lee et al. evaluated the outcomes of patients with BD-IPMN who had no significant changes within the first 5 years after diagnosis, showing that the mortality rate at 12 years for ACCI of ≤3, 4–6, and ≥7 were 3.5%, 19.9%, and 57.6%, respectively, and that the rate of non-pancreatic deaths was 97.5% of all deaths [31]. Based on these results, conservative management without undergoing surgical resection may be acceptable in patients with high ACCI without SC because of the low risk of IPMN-related death and the poor postoperative prognosis. The optimal cut-off value for the ACCI may vary in the primary disease and treatment. In most previous reports, the cut-off value for the ACCI was 4–8 [16,17,18,19]. The most appropriate cut-off value for the ACCI for IPMN exhibiting HRS has not been reported. In this study, based on the results of ROC analysis, a score of five or higher was defined as high ACCI.

**Figure 5:**
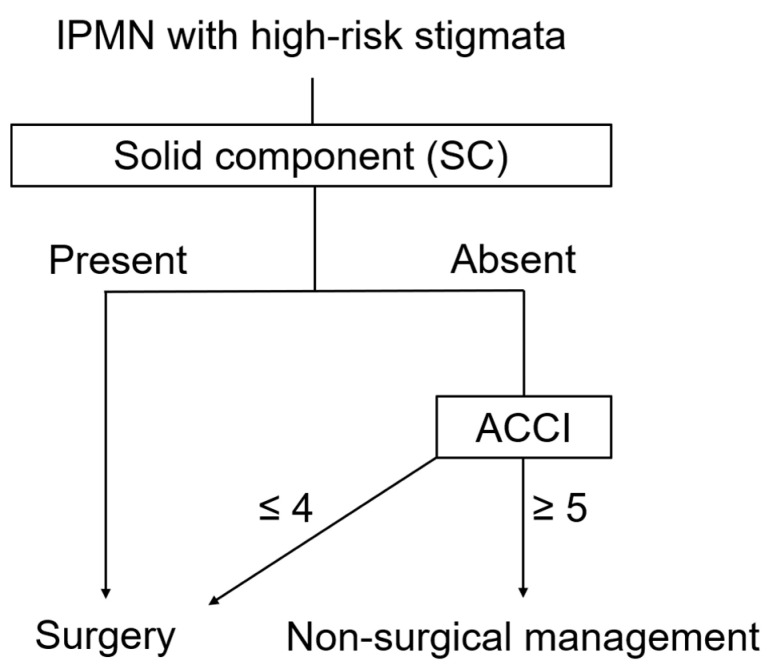
New treatment strategy for IPMN with high-risk stigmata. IPMN, intraductal papillary mucinous neoplasm; ACCI, age-adjusted Charlson comorbidity index.

On the other hand, some patients without SC at diagnosis developed SC during the long-term follow-up and died for reasons related to IPMN. Previous studies examining the natural history of IPMN with HRS reported that the IPMN-related death is 5.9–34% [10,29]. Even without SC, patients with HRS are at risk for IC in the long term. Therefore, patients with a low ACCI (≤4), such as young patients or patients without comorbidities, who are more likely to survive long-term should undergo surgery according to the Kyoto guidelines 2024 (Figure 5).

This study has several limitations. First, this study had a single-center retrospective design enrolling a limited number of patients. Thus, multicenter prospective cohort studies are required to better demonstrate the feasibility of the management in patients with IPMN exhibiting HRS. Second, the median follow-up period was less than 5 years. A longer period of observation is required to accurately assess patient prognosis. Third, preoperative pathology was not performed in this study. The usefulness of EUS sampling for SC and mural nodules has been reported, which may improve preoperative diagnostic performance [32,33]. Despite these limitations, this study is the first to compare the prognosis of patients with IPMN exhibiting HRS between the surgical and non-surgical groups. Furthermore, we determined that SC presence and ACCI may serve to establish more valid criteria for surgical resection than the conventional HRS.

## 5. Conclusions

In conclusion, the presence of an SC and a high ACCI are prognostic factors in patients with IPMN exhibiting HRS. Patients with an SC should undergo surgical resection because of the potential for an improved prognosis. Even without an SC, patients with a low ACCI (≤4) should be recommended for surgical resection due to the risk of future IC. However, in patients without an SC and with a high ACCI (≥5) (i.e., elderly or high-surgical-risk patients), conservative management may be the optimal treatment.

## Figures and Tables

**Table 1:** Clinical characteristics of the study patients.

	Surgical Group	Non-Surgical Group	*p* Value
	(*n* = 79)	(*n* = 36)	
Median age, years (IQR)	72 (65–77)	81 (77–83)	<0.001 *
Male, *n* (%)	51 (65)	22 (61)	0.88
Median ACCI (IQR)	4 (2–5)	5 (4–5)	0.001 *
MD/MIX type, *n* (%)	55 (70)	24 (67)	0.92
Median MPD, mm (IQR)	7 (3.8–11)	10 (3.8–11)	0.70
Median Mural nodule, mm (IQR)	7 (3.5–11.5)	6 (0–8)	0.18
Jaundice, *n* (%)	5 (6)	0	0.29
Solid component, *n* (%)	29 (37)	7 (19)	0.10
Death, *n* (%)	26 (32)	11 (30)	0.70
IPMN-related death, *n* (%)	12 (15)	7 (19)	
IPMN-unrelated death, *n* (%)	9 (11)	3 (8)	
Unknown, *n* (%)	5 (6)	1 (3)	
Pathological diagnoses			
low-grade dysplasia, *n* (%)	29 (37)		
high-grade dysplasia, *n* (%)	21 (26)		
IPMN-derived invasive carcinoma, *n* (%)	29 (37)		

* Statistically significant. IQR, interquartile range; ACCI, age-adjusted Charlson comorbidity index; MD, main duct; MIX, mixed; MPD, main pancreatic duct; IPMN, intraductal papillary mucinous neoplasm.

**Table 2:** Univariate and multivariate analyses of the predictors of overall survival (*n* = 115).

		Univariate Analysis			Multivariate Analysis		
		No.	5-Year OS (%)	*p* Value	HR	95% CI	*p* Value
Age	≥75	60	64	0.018	1.73	0.85–3.52	0.13
Sex	Male	73	63	0.68			
ACCI	≥5	41	55	<0.001	2.27	1.11–4.64	0.025 *
IPMN type	MD/MIX	79	68	0.35			
MPD	≥10 mm	53	64	0.65			
Mural nodule	≥5 mm	80	70	0.56			
Jaundice	Present	5	NA	0.028	2.03	0.56–7.43	0.28
Solid component	Present	36	35	<0.001	6.92	3.30–14.5	<0.001 *
Pancreatectomy	Yes	79	67	0.75			

* Statistically significant. OS, overall survival; HR, hazard ratio; CI, confidence interval; ACCI, age-adjusted Charlson comorbidity index; IPMN, intraductal papillary mucinous neoplasm; MD, main duct; MIX, mixed; MPD, main pancreatic duct; NA, not applicable.

## Data Availability

The authors declare that the data for this research are available from the correspondence author upon reasonable request.

## Supplementary Materials

The following supporting information can be downloaded at: https://www.mdpi.com/article/10.3390/diagnostics14222582/s1, Figure S1: Flow diagram of the study population; Figure S2: The receiver-operating characteristic (ROC) curves of ACCI for death; Figure S3: EUS and pathological findings of IC; Figure S4: Overall survival (OS) of patients without a solid component in the surgical group; Table S1: The causes of death among the study patients.
