# Supplementary material for: New Surgical Criteria for Intraductal Papillary Mucinous Neoplasm Based on the Age-Adjusted Charlson Comorbidity Index Values and Presence of Solid Component
Source: Diagnostics (Basel). 2024 Nov 17;14(22):2582. doi: 10.3390/diagnostics14222582 (PMC11592943; doi:10.3390/diagnostics14222582)
Supplement: Supplementary file 1 [file diagnostics-14-02582-s001.zip › Figure legends.pdf]

Figure legends

Figure S1. Flow diagram of the study population. IPMN, intraductal papillary mucinous neoplasm; HRS, high-risk stigmata; PDAC, pancreatic ductal adenocarcinoma.

Figure S2. The receiver-operating characteristic (ROC) curves of ACCI for death. The cut-off value is 5 (AUC, 0.635; sensitivity 0.331; specificity, 0.901). ACCI, age-adjusted Charlson comorbidity index; AUC, area under the curve.

Figure S3. EUS and pathological findings of IC. a) A case of IC diagnosed with an SC preoperatively. EUS showing an SC (arrow) in the pancreatic parenchyma that is contiguous with the mural nodule (arrowhead). Pathological result of cystic lesion indicates IC. IC invades to the stroma (black arrow). b) A case of IC diagnosed without an SC preoperatively. EUS showing the mural nodule (arrowhead) and no SC. Pathological result of slid lesion indicates IC with minimal invasion (black arrowhead). EUS, endoscopic ultrasonography; IC, IPMN-derived invasive carcinoma; SC, solid component.

Figure S4. Overall survival (OS) of patients without a solid component in the surgical group. The estimated 5-year OS in patients with a high ACCI (≥ 5) and those with a low ACCI (≤ 4) were 59% and 93%, respectively (P = 0.005). ACCI, age-adjusted Charlson comorbidity index.
