# Supplementary figures and images for: New Surgical Criteria for Intraductal Papillary Mucinous Neoplasm Based on the Age-Adjusted Charlson Comorbidity Index Values and Presence of Solid Component
Source: Diagnostics (Basel). 2024 Nov 17;14(22):2582. doi: 10.3390/diagnostics14222582 (PMC11592943; doi:10.3390/diagnostics14222582)

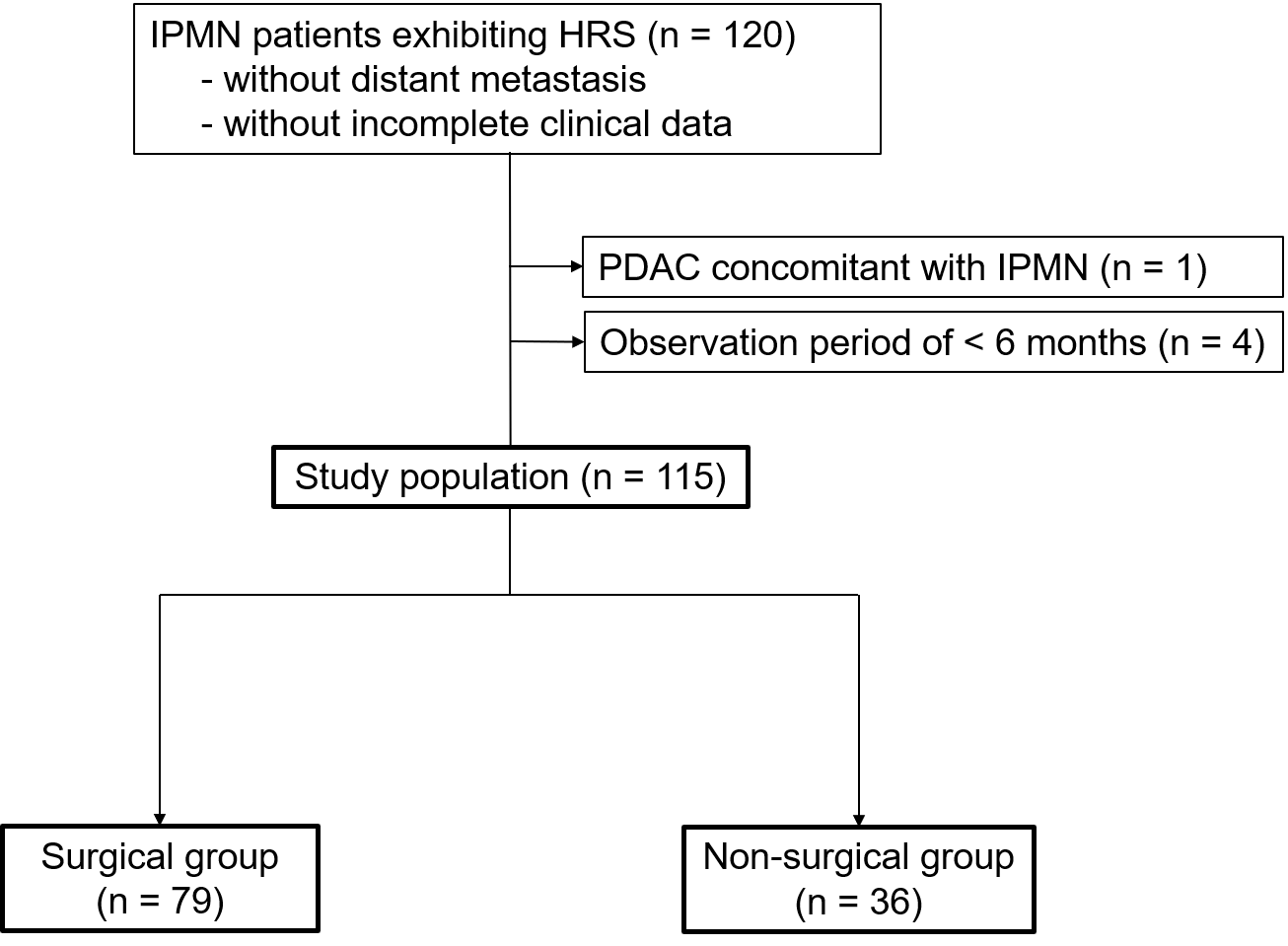

Supplement: Supplementary file 1 [file diagnostics-14-02582-s001.zip › Figure S1.tif]

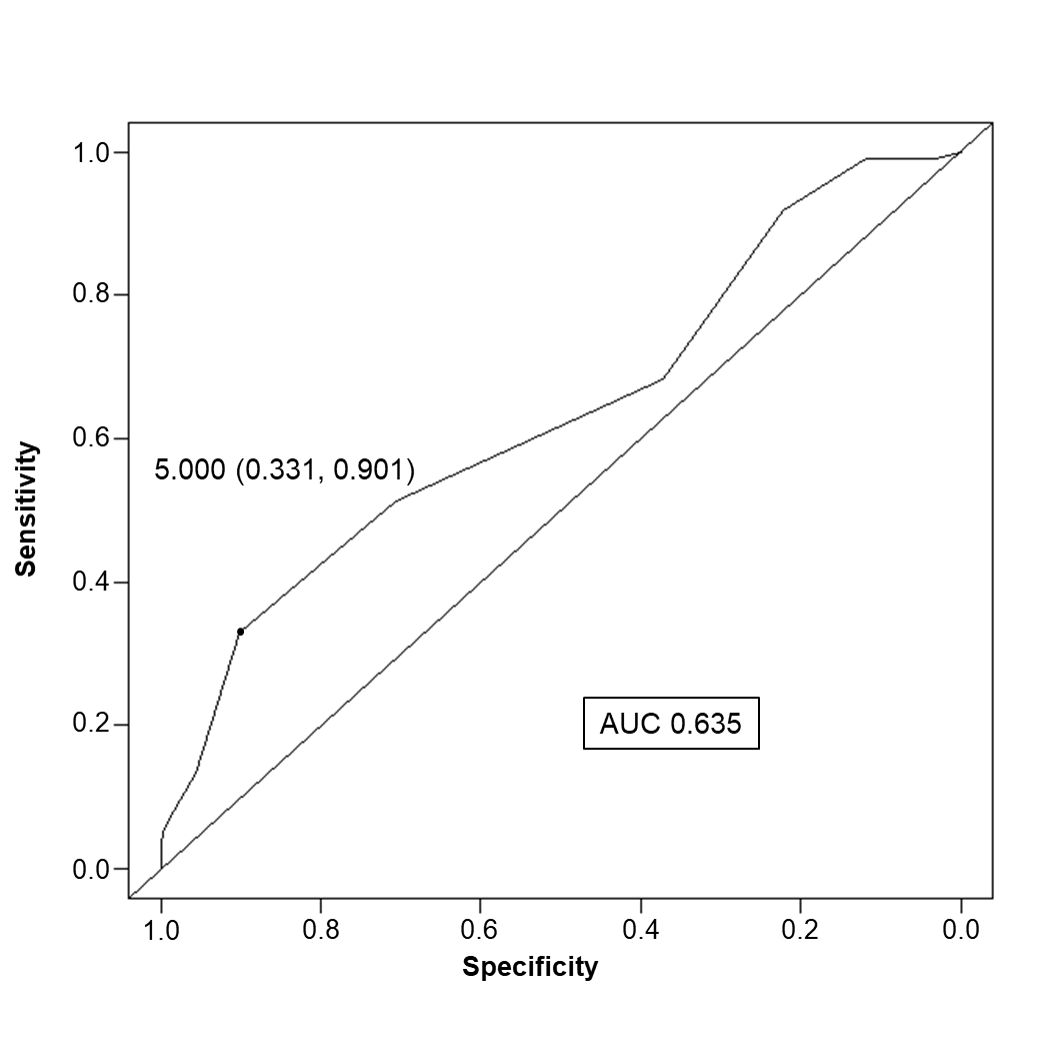

Supplement: Supplementary file 1 [file diagnostics-14-02582-s001.zip › Figure S2.tif]

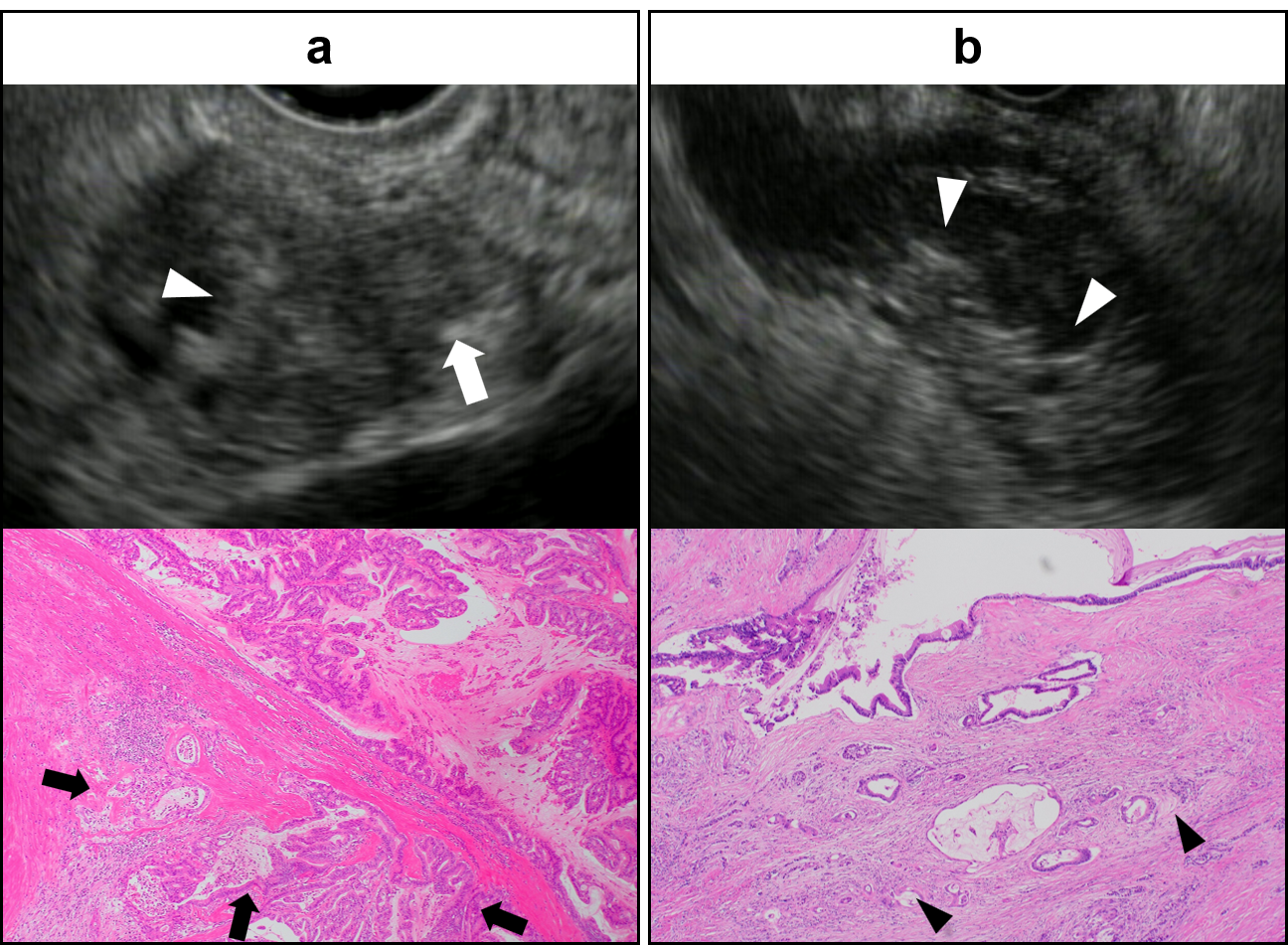

Supplement: Supplementary file 1 [file diagnostics-14-02582-s001.zip › Figure S3.tif]

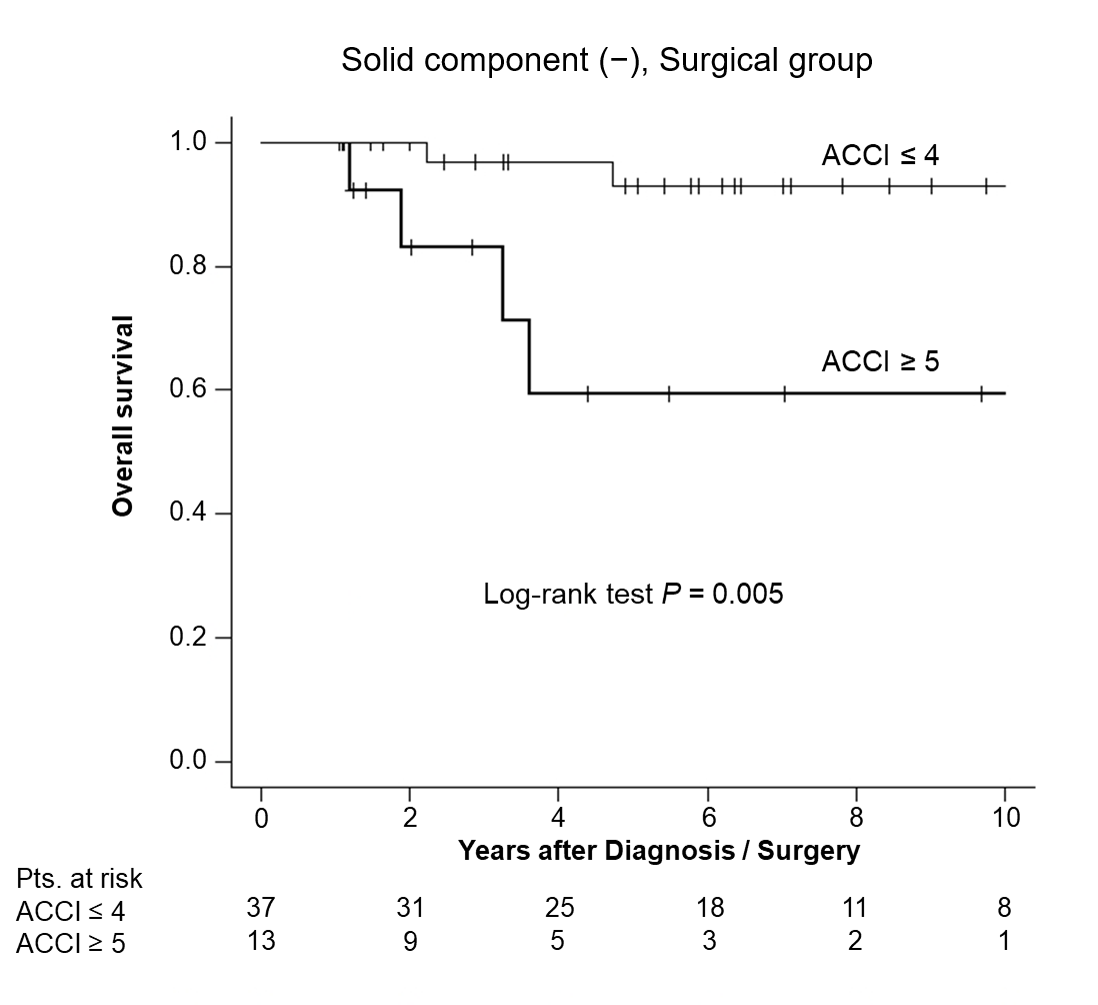

Supplement: Supplementary file 1 [file diagnostics-14-02582-s001.zip › Figure S4.tif]
